# Exploring the burden of cholera in the WHO African region: patterns and trends from 2000 to 2023 cholera outbreak data

**DOI:** 10.1136/bmjgh-2024-016491

**Published:** 2025-01-22

**Authors:** Etien Luc Koua, Fleury Hybriel Moussana, Vincent Dossou Sodjinou, Freddy Kambale, Jean Paul Kimenyi, Saliou Diallo, Joseph Okeibunor, Abdou Salam Gueye

**Affiliations:** 1 World Health Organization, Brazzaville, Congo; 2 Emergency Preparedness and Response, World Health Organization Regional Office for Africa, Dakar, Senegal; 3 World Health Organization, Dakar, Senegal; 4 Regional Office for Africa, Brazzaville, Congo; 5 Emergency Preparedness and Response, World Health Organization, Brazzaville, Congo; 6 Department of Sociology/Anthropology, University of Nigeria, Nsukka, Nigeria; 7 Emergency Preparedness and Response Programme, Brazzaville, Congo

**Keywords:** Cholera, Global Health, Control strategies, Epidemiology, Public Health

## Abstract

**ABSTRACT:**

**Introduction:**

Cholera outbreaks remain persistent in the WHO African region, with an increased trend in recent years. This study analyses actual drivers of cholera including correlations with water, sanitation, and hygiene (WASH) indicators, and climate change trends.

**Methods:**

This was a cross-sectional descriptive and analytic study. Cholera data from 2000 to 2023 and data relating to cholera drivers were compiled and analysed through multi-level exploratory analysis. We cross-referenced several WASH indicators, and generated a similarity matrix to categorise countries or subnational units into groups using principal component analysis and K-means clustering. We integrated cholera outbreak data with WASH indicators and created a matrix of indicators relevant for analysing cholera burden. We conducted summary statistics, temporal visualisations, Geographic Information System (GIS) mapping, trend analysis and statistical tests for correlations to derive patterns and trends from the data, derive similarities and develop projections.

**Results:**

A total of 2 727 172 cases and 63 182 deaths were reported from 44 countries, representing 94% of the 47 countries in the region, from 2000 to 2023. The case fatality ratio of 2.3% is suggestive of issues in case management. A total of 684 outbreaks were reported, with the highest burdens in Nigeria and the Democratic Republic of the Congo. Median detection time to outbreak was 2 days, while median time for outbreak control was 92 days. Cholera incidence seemed higher in the period 2014 to 2023 than in the period before 2014. The study results confirmed correlations between WASH indicators and cholera outbreaks. Risks factors include drinking surface water, lacking soap and/or water, and open defaecation. Over 29% and 58.8% of the population lack access to basic water and basic sanitation, respectively.

**Conclusion:**

Insufficient access to WASH services remains the main predisposing factor for cholera in the WHO African region. Political leaders should invest more in access to WASH, strengthen multisectoral collaboration, and improve availability of needed tools to increase the likelihood of meeting cholera elimination goals by 2030.

**WHAT IS ALREADY KNOWN ON THIS TOPIC**

- There is some debate about the most important factors currently driving cholera outbreaks in the WHO African region.
- Factors proposed as important involve those resulting in bringing people and disease-causing organisms closer, such as climate change, but poor water and sanitation infrastructure are also thought to be important.

**WHAT THIS STUDY ADDS**

- We analysed 24 years’ worth of data for 2000–2023, which clearly reveal improved early detection of outbreaks, reporting of outbreaks from hotspots, long lag times to control, and increased incidence from 2014 to 2023.
- The analysis reveals that slow progress in the development of good water, sanitation, and hygiene (WASH) infrastructure and the associated challenges remain the key factors for cholera outbreaks in the African region.
- Development of these infrastructures has been much slower and not commensurate with population growth and demands for WASH in the region.
- Very marginal improvement in access to basic WASH infrastructure has been witnessed in the region between 2015 and 2022.

**HOW THIS STUDY MIGHT AFFECT RESEARCH, PRACTICE OR POLICY**

- Our analysis provides evidence which might help project ahead towards 2030 to help guide managers and policymakers.
- All countries and areas within countries should be prepared for the need to respond to cholera outbreaks.

## Introduction

 Cholera outbreaks in the WHO African region continue to evolve with an increasing number of cases and deaths. In 2021, the region experienced its first most severe and deadly outbreak in decades with a total of 137 125 cases and 4065 deaths reported.^1 2^ This situation was driven by the unprecedented high burden from cholera outbreaks reported in West Africa that led to at least 108 859 cases and 3711 deaths.^2^ Since 2021, the number of countries affected have increased, leading to 193 000 cases reported in 2023.

The main predisposing factors for cholera remain the lack of access to adequate water, sanitation, and hygiene (WASH). Reports reveal that 418 million people lack safe drinking water, 779 million lack basic sanitation and 839 million lack basic hygiene services in Africa,^3^,^5^ undermining efforts to end extreme poverty and disease in the region. Sustainable Development Goal (SDG) 6 is to ‘ensure availability and sustainable management of water and sanitation for all’. The targets cover all aspects of both the water cycle and sanitation systems, and their achievement is designed to contribute to progress across a range of other SDGs, notably on health, education, economics, and the environment. With the slow progress made so far, it is expected that the region will not meet the 2030 Agenda for SDG target 6, further exposing the region to more water and sanitation related public health threats including cholera. In fact, in many countries, open defaecation is one of the major drivers of cholera outbreaks.^6^ The disease is linked to SDG 6 as the first and second targets of SDG 6 are related to access to safe water (target 1) and adequate sanitation and hygiene services (target 2). On the other hand, recent studies have suggested that climate change is exacerbating the risk for infectious diseases by bringing people and disease-causing organisms closer together.

This is the case for cholera. For example, one study^3^ has investigated how surging temperatures, sea level rises and droughts have affected all documented infectious disease spread. Other studies have found that increases in temperature and rainfall have expanded the range of mosquitoes and contributed to outbreaks of dengue fever, chikungunya, and malaria. Similarly, heatwaves draw people to water-related activities, leading to a rise in cases of waterborne illnesses; storms, sea level rise and floods force people to move and have been implicated in outbreaks of Lassa fever, cholera and typhoid fever.^7 8^

In one study exploring droughts and floods and their association with cholera outbreaks in sub-Saharan Africa, it has been noted that a cholera outbreak was registered in one of every three droughts and one of every 15 floods. The study observed an increased incidence rate of cholera outbreaks during drought periods (incidence rate ratio (IRR) 4.3, 95% confidence interval (95% CI) 2.9 to 7.2) and during flood periods (IRR 144, 95% CI 101 to 208) when compared with drought/flood-free periods. According to the authors, floods are more strongly associated with cholera outbreaks, yet the prevalence of cholera outbreaks is higher during droughts because of their long duration.^9 10^ These findings need validation in areas not yet known as prone to cholera as well as prolonged and protracted outbreaks reported in the regions since 2021. Additionally, conflict, natural disasters, and cross-border movements are also known to act as driving factors for outbreaks within the region, including cholera. Despite these known risk factors, the recent trends of cholera outbreaks in the region are concerning. After the biggest cholera outbreak reported in west Africa, eastern and southern Africa is experiencing a high burden of protracted cholera outbreaks. Malawi has experienced cholera outbreaks for close to 2 years. Areas not previously thought to be vulnerable to cholera are being affected and the case fatality ratio is thought to be high. It is not clear whether currently implemented control interventions there are reaching their objectives. If the trends described above are sustained, the region will not reach its objectives for elimination by 2030, per the regional framework for implementation of the global strategy for cholera elimination by 2030, endorsed in 2018 by African member states. Adequate corrective actions to change this situation can only be informed by authoritative data analysis to generate evidence. Therefore, we aimed to use 24 years of data on cholera in the African region to review the known factors, using a diversity of data sources, and to better inform preparedness and response to cholera in the region.

## Settings and methods

### Setting

The study is conducted in the WHO African region, one of six WHO regions across the world. The African region covers 47 member states, of which at least 12 are affected by cholera outbreaks each year. The disease is endemic in some countries while it displays an epidemic mode in others. Almost all the countries in the region have experienced cholera outbreaks at least once since the disease first reached the region during the seventh cholera pandemic in 1971.^9^ In August 2018, the region’s member states endorsed a regional framework for the implementation of the global strategy for cholera elimination 2018–2030. This framework sets out clear milestones to be achieved by countries to eliminate cholera by 2030.

### Methods

This was a cross-sectional descriptive and analytic study. The overall scope of this work was to compile and analyse available data at national and subnational levels on routine surveillance, cholera outbreaks and related factors such as WASH.

We used an exploratory analysis method that has several levels of analysis. The first level of this analysis consists of cross-referencing several indicators relevant to WASH issues to provide an understanding of patterns across these indicators. A similarity matrix is then used to categorise countries or subnational units into groups using principal component analysis (PCA) and K-means clustering. A second level of the analysis consists of integrating cholera outbreak data with WASH indicators and creating a matrix of indicators. We then determined which indicators are most relevant for analysing cholera burden and the most left-behind populations, based on pattern analysis. The analysis integrates some level of multidimensional scaling to come up with classification of countries based on cholera burden and WASH indicators. Summary statistics, temporal visualisations, Geographic Information System (GIS) mapping, trend analysis and statistical tests for correlations are used to derive patterns and trends from the data, derive similarities and develop projections. A further geographic analysis is used to visualise (1) available WASH data, (2) relevant seasonal factors (eg, flooding), (3) comparing previous and current coverage of WASH, (4) distribution of water-borne diseases including cholera, (5) population density, and (6) relevant sociodemographic and (7) ecological characteristics in order to identify ‘hot spots’.

## Data sources

Several datasets are used for the analysis including routine integrated disease surveillance and response (IDSR) data, historical cholera events management data, WASH data, and other sociodemographic and economic data.

- Cholera outbreak dataset extracted from WHO Global Health Observatory data repository which contains data validated by countries and published in cholera annual report at global level.^10^
- WHO AFRO public health events documented since 2000, containing public health events for which a formal notification has been issued by public health authorities in the region. The number of cholera outbreaks was recalculated with other sources and may be underestimated as many outbreaks reported in countries per year were not all recorded in the WHO Event Management System.
- The WHO/UNICEF Joint Monitoring Programme global data on WASH.^11^ This dataset includes three main service types (sanitation, hygiene, and drinking water) at national and subnational level for each country. The data source will help build a heatmap of highest burden areas and most left behind populations focusing on WASH indicators. The dataset has a measure of quality depending on a calculated coverage (eg, basic service, at least basic, limited service, unimproved, open defaecation, and safely managed for sanitation).
- IDSR dataset that records suspected cases and deaths of various diseases every week at district level for each country in the region.^12^

### Data analysis

Data cleaning and analyses were performed using Microsoft Excel, R software version 4.1.3, and ArcGIS Desktop. The units of analysis were countries and districts. We review cholera epidemiological data completeness and proceed to cleaning using Microsoft Excel. Data were inserted in R software version 4.1.3 for analysis. The count of outbreaks was based on the WHO Event Management System data. WASH data were analysed with R software. ArcGIS Desktop was used to develop maps.

Several levels of analysis were engaged in line with the exploratory analytic design of this study. The first level of analysis entailed the underlining and cross-referencing of some critical WASH indicators that provide clarity of patterns across these indicators. A similarity matrix was then employed to categorize countries or subnational units into groups using PCA and K-means clustering.

The second level of the analysis involved integrating cholera outbreak data with WASH indicators to generate a matrix of indicators for identifying the most appropriate for analyzing cholera burden. The analysis integrated some level of multidimensional scaling to produce classification of countries based on cholera burden and water, sanitation, and hygiene indicators. Descriptive statistics, temporal visualizations, GIS mapping, trend analysis and statistical tests for correlations were used to derive patterns and trends from the data, derive similarities and develop projections. Furthermore, geographic analysis was used to visualize (a) available WASH data, (b) relevant seasonal factors (e.g., flooding), (c) comparing previous and current coverage of WASH, (d) distribution of water-borne diseases including cholera, (e) population density, and (f) relevant socio-demographic and (g) ecological characteristics to identify “hot spots”.

## Results

### Trends of cholera outbreaks from 2000 to 2023

**Figure 1:**
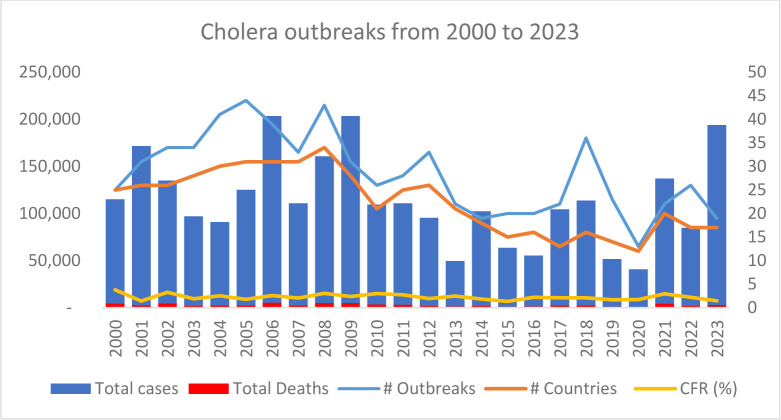
Trends of cholera outbreaks from 2001 to 2023, with number of cholera outbreaks and number of affected countries. CFR, case fatality rate

The WHO African region recorded on average 28 cholera outbreaks per year since 2000 with an average of 113 632 cases per year and 2633 deaths every year. A total of 2 727 172 cases and 63 182 deaths were recorded from 2000 to 2023, an average case fatality rate (CFR) of 2.3%. Almost every country (44 out of the 47 countries in the WHO African Region) has experienced a cholera outbreak since 2000 (online supplemental table 1). Figure 1 presents the trend of cholera outbreaks from 2000 to 2023, with the number of cases, deaths, and number of affected countries.

A summary of the statistics of cholera outbreaks from 2000 to 2023 is presented in online supplemental table 1. The statistics also show multiple cholera outbreaks the same year in the same country, pointing to the challenges presented by cholera in the region.

Eight countries, namely Democratic Republic of the Congo (DRC), Nigeria, Mozambique, Zimbabwe, South Africa, Ethiopia, Angola, and Malawi had the highest burden of cholera with more than 100 000 cases in the period 2000–2023. Uganda, Nigeria, Zimbabwe, DRC, Kenya, Burundi and Mozambique had more than 20 cholera outbreaks. DRC reported the greatest number of cases (588 996; 24%) and deaths (13 877; 22%) over the period and is one of the countries which reported the greatest number of cholera outbreaks (24) with relatively high incidence and mortality rate, but with moderate lethality. Nigeria also reported a high number of outbreaks (29) but had a lower mortality rate than the DRC (4.8 vs 13.6), which would indicate a potentially better case management system. Even though South Africa is one of the top five countries having reported the most cases, the death rate per 100 000 people (0.9) and the lethality (0.35) are among the lowest recorded, potentially suggesting better management for cholera cases. Online supplemental table 2 provides more details on cholera burden by country.

**Figure 2:**
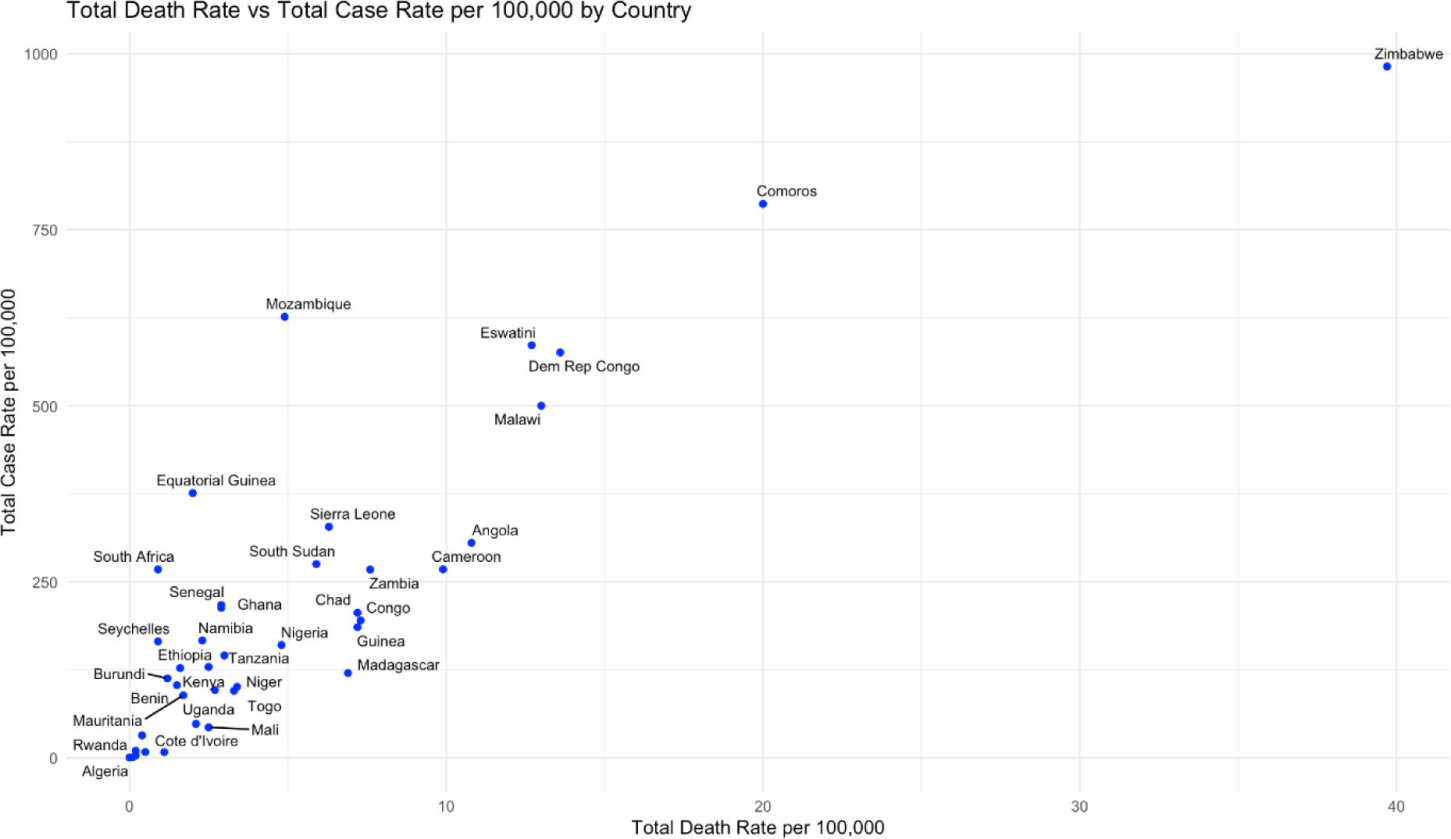
Total case rate and death rate.

**Figure 3:**
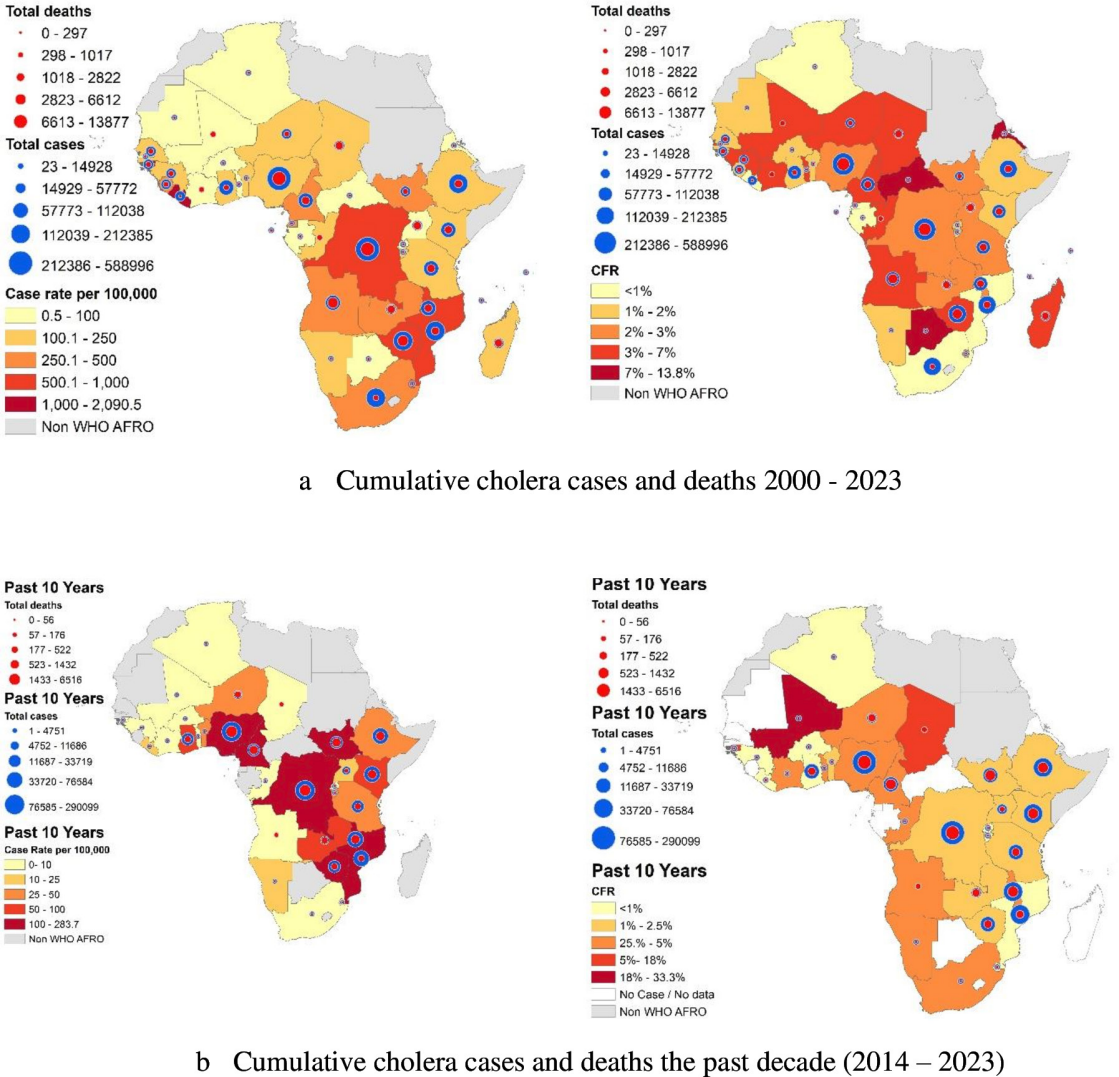
Map of cholera outbreaks, cumulative cases, deaths, and CFR (2000–2023). CFR, case fatality rate.

As depicted in figure 2, Zimbabwe and Comoros had the highest case incidences, death rate per 100 000 population and case fatality, suggesting significant challenges in managing cholera outbreaks in these countries; Algeria and Rwanda had the lowest rates. In addition to the summary statistics, the maps in figure 3 present the cumulative cases and deaths of cholera outbreaks from 2000 to 2023, to help explore the geographic patterns of cholera outbreaks. DRC (588 996 cases; 13 877 deaths) and Nigeria (358 774 cases; 10 817 deaths) were the top two of countries in terms of cases and deaths and reported respectively 24 and 29 outbreaks. Eritrea (120 cases; nine deaths) and Botswana (23 cases, three deaths) were the two countries with the lowest burdens, with two outbreaks reported each during this period (online supplemental table 2).

From 2000 to 2023, the highest cholera incidences were reported in central Africa (DRC and surrounding countries), in eastern Africa around Mozambique, and in west Africa mainly in Lake Chad basin (Chad, Cameroon, Niger, Nigeria). The highest CFRs were reported in west Africa and in central Africa. Compared with the overall period (2000 to 2023), in the last 10 years (2014 to 2023) the incidence of cholera seems higher in eastern Africa, central Africa (Cameroon, Central African Republic, DRC) and in western Africa (Ghana, Niger, Nigeria) including in Lake Chad basin. However, the CFR trend was in higher in the period 2014 to 2023 than in the overall period (2000 to 2023) (figure 3).

**Figure 4:**
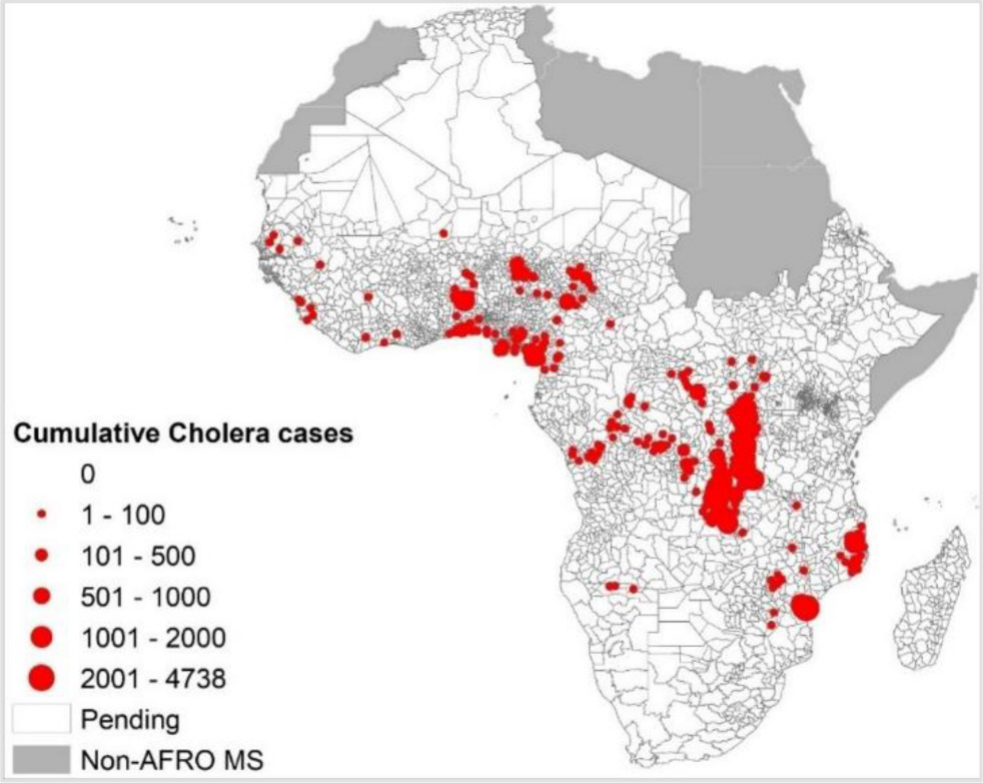
Pattern of cholera disease burden using cumulative IDSR data of suspected cholera cases from 2019 to 2023. IDSR, integrated disease surveillance and response; non-AFRO MS,Non Member States of the WHO African Region.

The map in figure 4 shows a concentration of cholera outbreaks in the centre-eastern part of the African region. To understand better the geographic patterns, we use IDSR data 2019–2023 to analyse the district level data. Figure 4 shows the concentration of suspected cholera cases at district level. This excludes data from the two countries Algeria and South Africa, which did not adopt IDSR. South Africa has recently adopted IDSR and is currently in the process of implementing data collection tools for weekly IDSR data collection. The trend of suspected cases is superimposable to outbreaks data in central and west Africa.

### WASH and cholera disease burden

An analysis of the WASH situation in the region was conducted based on indicators and internationally comparable estimates of progress on WASH produced by the WHO/UNICEF Joint Monitoring Programme for WASH. Indicators included in the analysis are presented in online supplemental table 3.

**Figure 5:**
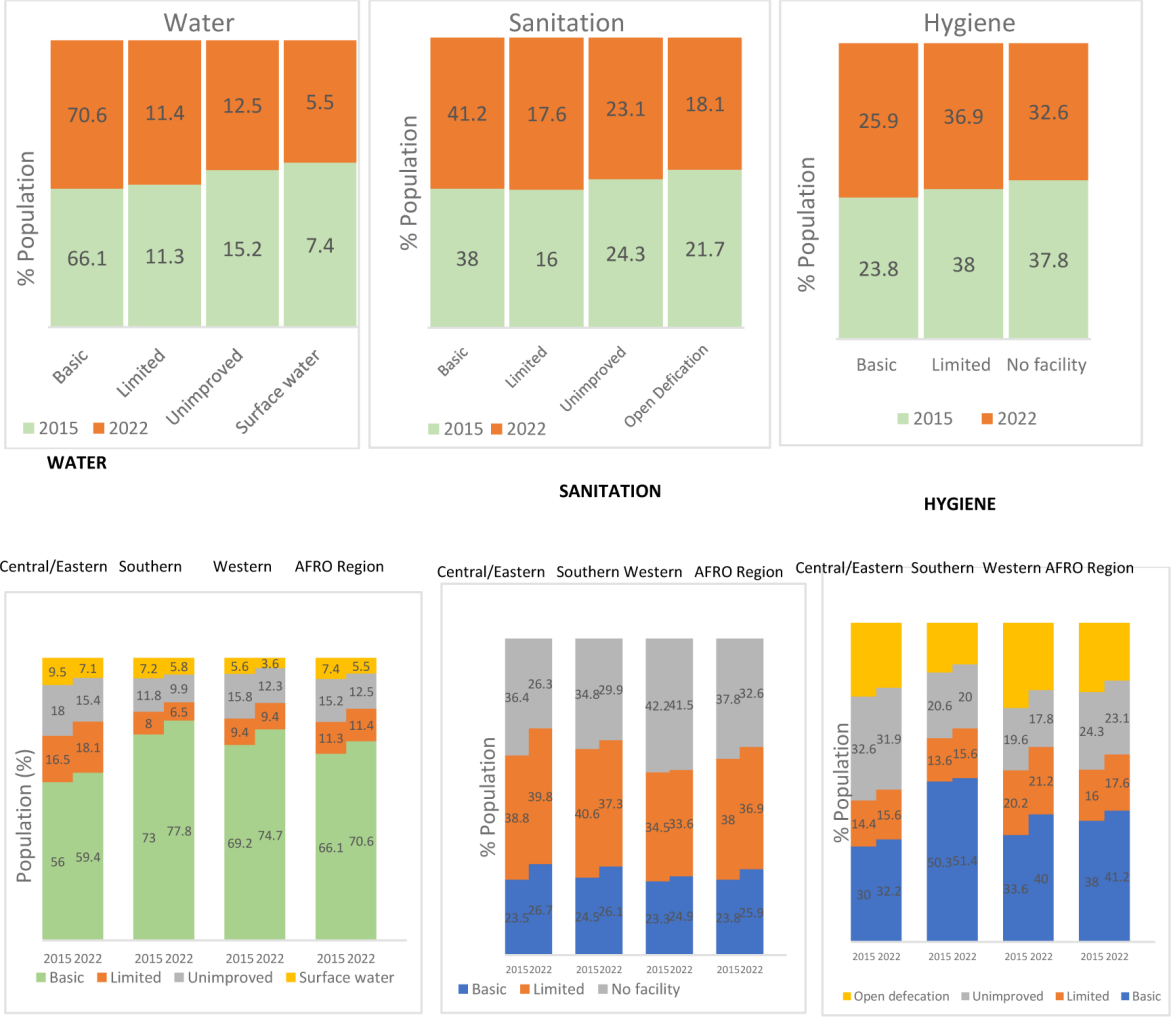
Water, sanitation and hygiene coverage in the WHO AFRO region and sub region, 2015–2022.

In the WHO African region, 41.2% of the population used basic sanitation, and 25.9% used basic hygiene in 2022 (figure 5). There is a slow progression between 2015 and 2022 (8 years), going from 38% to 41.2% for sanitation and 23.8% to 25.9% for hygiene. With current rates of progress, a dramatic acceleration is required to meet universal coverage (>99%) by 2030. Although access to safe drinking water has improved from 66% to 70.6% between 2015 and 2022, 30% of the population still drinks water from an unprotected source (figure 5).

In 2022, the population of Africa reached 1.43 billion, revealing substantial challenges in access to basic WASH facilities. Our analysis has revealed that:

- Approximately 415 million people, representing 29% of the population, lack access to basic water facilities.
- Between 2015 and 2022, the proportion of the Africa’s population that have access to basic drinking water increased from 66% to 71%.
- Over 800 million people, representing 58.8%, lack access to basic sanitation.
- The proportion of the African population that had access to basic sanitation increased from 38% to 42% from 2015 to 2022—representing only 46 million more people since 2015.
- More than 1 billion people, accounting for 74.1%, are living without basic hygiene facilities.
- More than 259 million people still practice open defaecation in 2022.

With the current trajectory, seven out of 10 individuals in Africa lack basic hygiene facilities, while six out of 10 lack basic sanitation facilities. The rate of change from 2015 to 2022 stands at 4.5%. Achieving universal coverage (>99%) by 2030 necessitates a significant escalation in progress rates, aiming for a 13-fold increase for basic water facilities and over a 40-fold increase for basic sanitation.

#### Projected scenarios by 2030

By the SDG target 2030, the African region will present the following deficit:

- About 360 million people will still lack access to basic water facilities.
- Over 1 billion individuals will continue to lack basic hygiene facilities.
- Equally concerning, 795 million people are at risk of having no access to basic sanitation if the current rate of change is not accelerated.

The WASH situation is very similar across the three sub regions in the WHO African region (central and eastern, southern and western Africa)

### Classification of countries based on their WASH profile

An analysis of the WASH indicators using K-means clustering technique revealed three main clusters, aligning with countries profiles.

*Cluster 1* represents countries with better access to water and sanitation (high values for the basic access to water and sanitation variables and low values for the unimproved and limited WASH variables, low open defaecation, and low use of surface water for drinking). Countries in this cluster include Algeria, Botswana, Mauritius, Cape Verde, Seychelles, and South Africa.

*Cluster 2* is characterised by limited access to WASH facilities. Example of countries included in this cluster are Cameroon, Congo, Gambia, Ghana.

**Figure 6:**
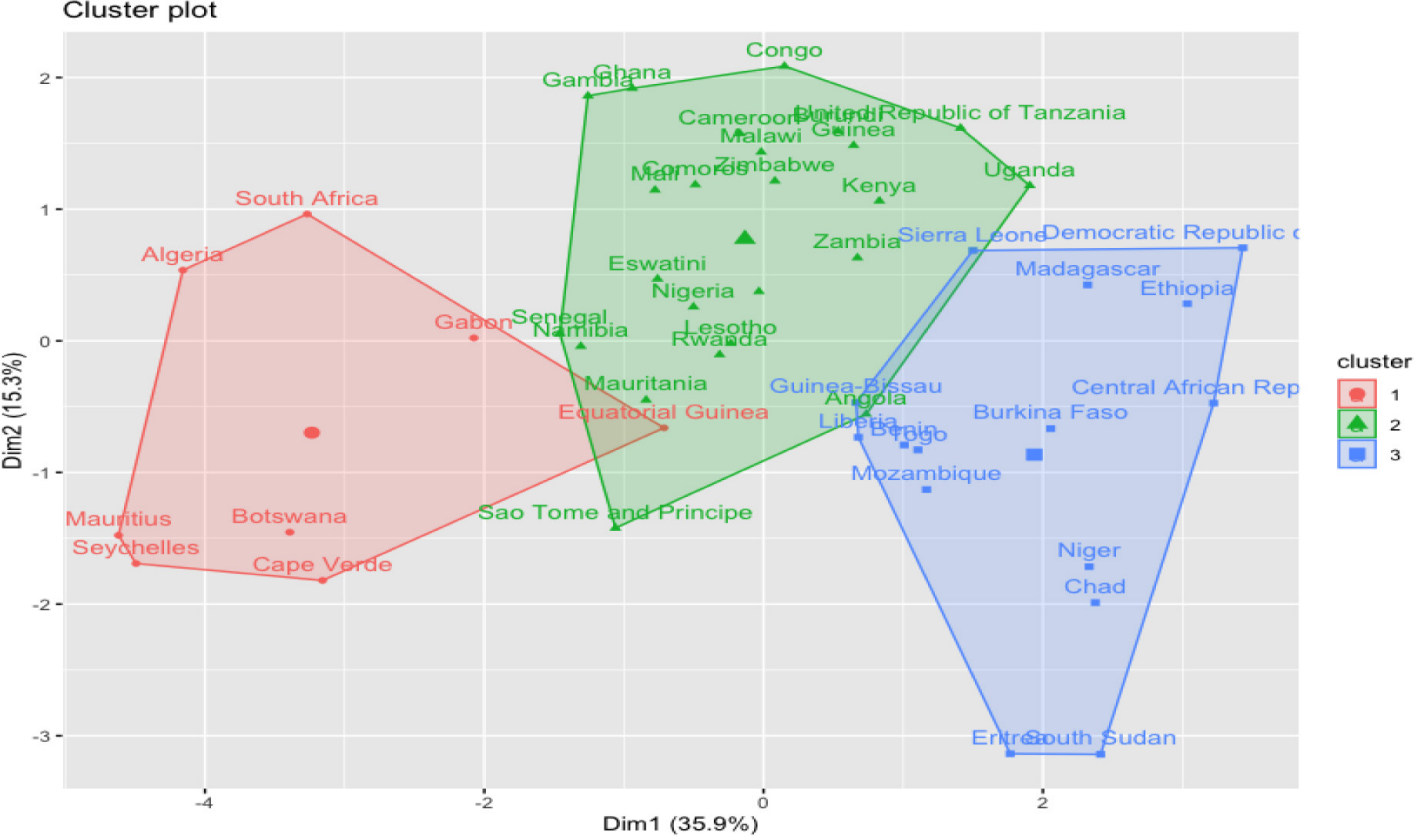
Clustering of countries based on water, sanitation, and hygiene indicators.

*Cluster 3* is characterised by lowest values for access to WASH services, with the highest proportion of open defaecation, and drinking of surface water. Countries in this cluster include Burundi, Central African Republic, Chad, DRC, Eritrea, Ethiopia, Madagascar, Niger, South Sudan, and the United Republic of Tanzania. The graphical representation of the clusters is presented in figure 6.

### Correlations between WASH and cholera disease outbreaks

We investigated correlations between WASH indicators and outbreak for the period 2015 to 2023. This is the period for which WASH data were available (2015 to 2022). Several correlations were revealed at 95% of significance level (online supplemental table 4).

- As anticipated, a positive correlation between cholera outbreaks and the proportion of populations using surface water was revealed. It shows that an increase in the proportion of the populations’ drinking water coming directly from unprotected sources increases the number of cholera outbreaks.
- There is also a positive correlation between cholera outbreaks and the proportion of population with limited hygiene (without water or soap), suggesting that an increase in the proportion of the populations without access to handwashing facilities at home will result in an increase in the number of cholera outbreaks.
- There is an inverse correlation between the number of outbreaks and the proportion of the population with at least basic water service, suggesting that access to basic water service leads to a decrease in the number of cholera outbreaks.

**Figure 7:**
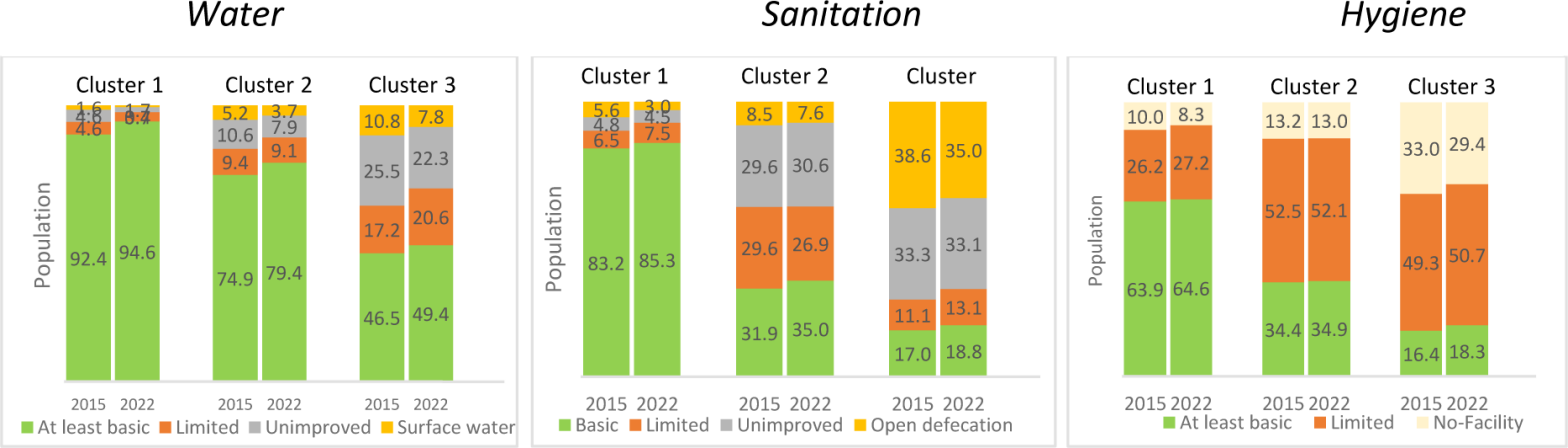
Water sanitation and hygiene profile for the different clusters.

The correlations are further illustrated in figure 7, showing WASH profiles for the different clusters identified, with cluster 1 showing better service coverage for WASH than cluster 2 and cluster 3. Cluster 3 shows most limited or no access to these services.

**Figure 8:**
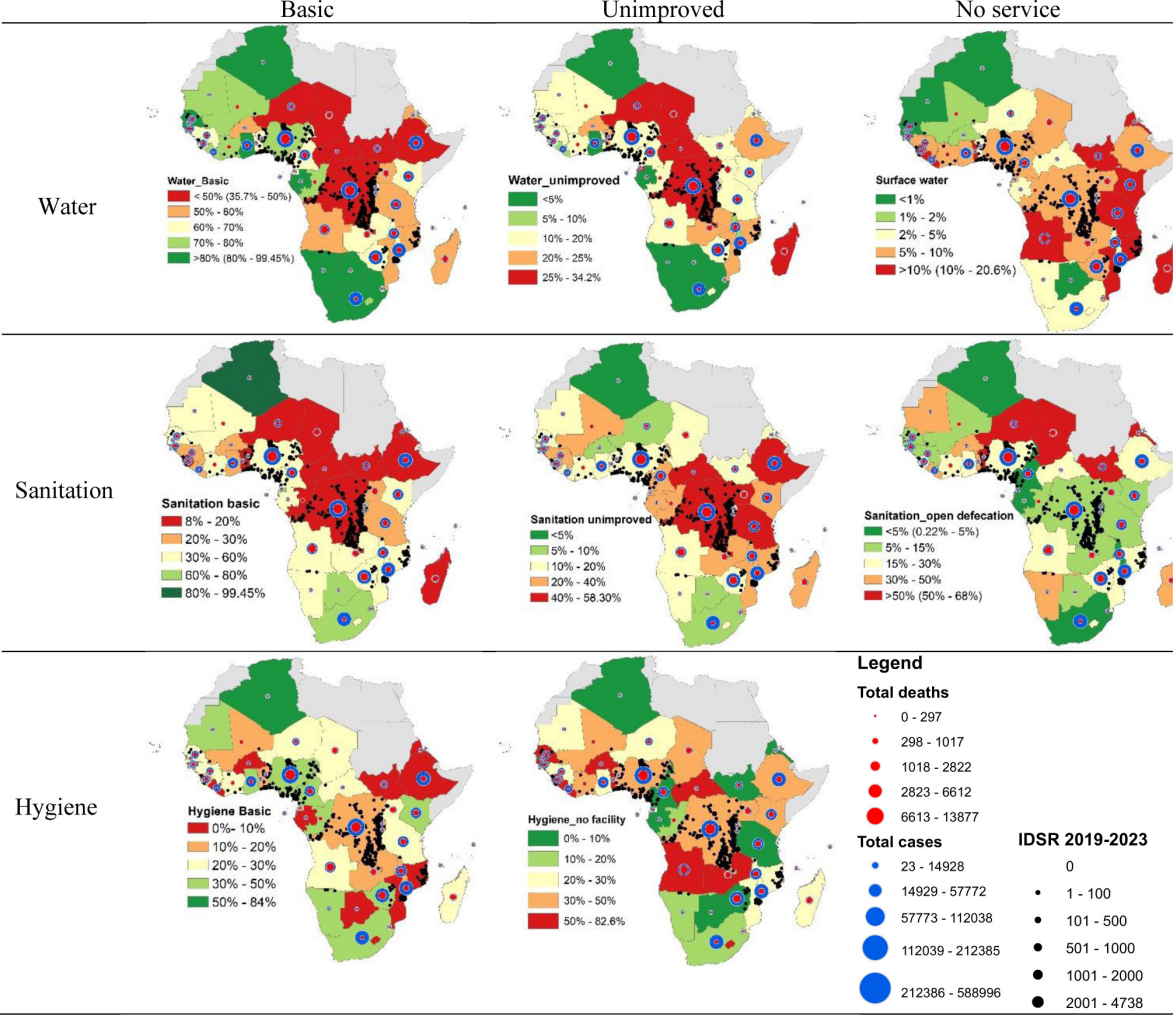
Water sanitation and hygiene profile for the different clusters. IDSR, integrated disease surveillance and response.

A geographic representation is also provided in figure 8 illustrating the countries and their WASH profiles as well as the cumulative total cholera cases and deaths for the entire period of study (2000–2023).

The map of access to WASH is superimposable to the map of cumulative case and death from 2000 to 2023. DRC and surrounding countries and Lake Chad basin are characterised by low access to basic water services (<50%), a high proportion of the population using unimproved water services (>25%) while in Mozambique and surrounding countries, <60% of the population have access to basic water service, 10% to 25% of the population are using unimproved water service, and >10% of the population are using surface water. The trend is the same for sanitation and hygiene practice. Areas with insufficient WASH are the same with high burden cholera outbreaks.

### Cholera surveillance

By 2002, 33 countries out of the 47 in the WHO African region had integrated cholera in their IDSR reporting (online supplemental table 5). It is noticeable that cholera outbreaks have occurred in many countries that do not have cholera integrated in their surveillance system at some point (Cameroon, Central African Republic, Congo, Eritrea, Gambia, Guinea Bissau, Kenya, Lesotho, Malawi, Mauritania, Niger, Rwanda).

From 2019 to 2023, a cumulative number of 16 countries reported inconsistently cholera suspected through IDSR. The annual number of countries varies from five in 2022 to 12 countries in 2021.

### Time to detection and time to contain a cholera outbreak

#### Regional level

Eighty-five of the 110 substantiated outbreaks (2015–2023) had a date of onset of symptoms and detection date. The overall median time to detection was 2 days (IQR 0–7), and the fastest detection was achieved in 2019 (0 day (IQR 0–3)). Most of these outbreaks were detected within 7 days. The overall median time to contain a cholera outbreak was 92 days (IQR 46–181). The fastest outbreak containment was achieved in 2018 after 49 days (IQR 25–86). Online supplemental table 6 provides details.

#### Detection and response time by country

The outbreaks have been mostly detected the same day in six countries, including Burundi, Cameroon, Congo, Ethiopia, Kenya, and Zimbabwe. The overall median time to detection was higher in four countries: Ghana 12 days (IQR 12–12), Algeria 17 days (IQR 17–17), Nigeria 19 days (IQR 9–28), and Angola 29 days (IQR 28–29).

Two countries quickly contained outbreaks: Namibia 6 days (IQR 6–6), and Cape Verde 8 days (IQR 8–8). Some countries detected the outbreak as early as possible but it took a very long time for control: Congo 100 days (IQR 100–100), Cameroon 534 days (IQR 471–594), and Ethiopia 753 days (IQR 753–753). Detailed countries’ data on detection and response time are provided in online supplemental table 7.

## Discussion

Cholera reached the WHO African region (AFRO) in the 1970s with a highly deadly trend in some countries for decades.^9^ With progressive improvement of health system capacity in the region, citizens should expect application of adequate measures to mitigate the burden of cholera, particularly deaths. However, some of the findings of this study raise some concerns.

### Cholera trends since 2014

From 2014 to 2023, the burden of the disease has increased with the occurrence of unprecedented high burden outbreaks since 2021.^2^ The recent trend of the disease is comparable to the trend observed from 2000 to 2010 while the number of reported outbreaks has improved (figure 1). This increase can be explained by the improvement of surveillance systems across the region. In fact, since the 2014 Ebola virus disease in west Africa, AFRO improved the implementation of the detection, verification and alert interventions aiming at strengthening early detection of diseases. This early detection is evidenced by the regional overall median time of cholera outbreak detection of 2 days (0–7) from 2015 to 2024. This improvement has also been seen for other diseases such as a study of 24 diseases for which the median time to detection decreased from 14 (IQR 6–37) days in 2017 to 7 (IQR 1–27) days in 2018 and to 4 (IQR 1–11) days in 2019 for 184 outbreaks of 24 diseases.^13 14^ AFRO also improved the implementation of the international health regulation notification through IDSR.^15^

On the other hand, many countries in the past were reluctant to notify cholera for many reasons and reported the disease as acute watery diarrhoea to WHO. Successful advocacy undertaken towards these countries has, since 2018, led to notification of cholera when confirmed. This increased trend can then be seen as evidence of health system capacities across the region in detecting and notifying outbreaks. The increased trend of the disease since 2014 can also be explained by the occurrence of cholera outside known hotspots in countries and in countries that reported cholera many years ago. The recent cholera outbreak in Comoros occurred 17 years after the last outbreak reported in 2007. By spreading outside the hotspots, the disease met unprotected and more susceptible populations, leading to high attack rates of the disease as reported in west Africa in 2021 and in eastern and southern Africa since 2022. The spread outside hotspots also met unprepared health systems with a delay in detecting the disease and a weakness in adequate management that could explain the high number of deaths. Preparedness for cholera should further go beyond known hotspots and embrace all countries and districts. This is highly needed as the recent outbreaks were characterised by cross border spread as the result of high mobility of the population in the region.^16 17^

### Time to control outbreaks

The failure in ensuring access to safe water and adequate sanitation, even during cholera outbreaks, can explain the long duration to control epidemics. The regional median time to control 78 cholera outbreaks from 2015 to 2023 was 92 days, ranging from a minimum of 46 days to a maximum of 181 days (online supplemental table 5). Although the situation varies from one country to another and from one outbreak to another, this long time to control outbreaks is concerning. A similar trend was previously reported^15^; this study analysed data for outbreaks of 34 diseases and found a reduction in time taken for control from 131 days in 2017 to 45 days in 2019.

The situation is also due to continued spread of outbreaks into new areas, leading to a series of new local outbreaks that prolong the overall time to control. Inadequate infrastructure (cholera treatment centres, cholera units, oral rehydration places) to manage cases and failure in compliance with infection prevention and control measures play a critical role; as well, the absence of a holistic response approach that includes an adequate preparedness component in at-risk areas around the affected settings contributes to the long time to control outbreaks. All these are influenced by the absence of sufficient and predictive funding to organise preparedness and response to cholera. But, as changes in access to WASH are slow and improved access to WASH is not realisable now, there are needs to seek for alternatives to timely control cholera outbreaks. Countries’ stakeholders and partners should strengthen preparedness to cholera, and plan and implement timely WASH emergency interventions such as adequate distribution of Aquatabs, water trucking, establishment of chlorination points in key areas and deployment of mobile latrines. During cholera outbreaks, governments should take necessary action to make free the access to water for vulnerable populations. The international community should also facilitate distribution of needed tools for prevention and control of cholera.^18^

### Outbreak correlation with WASH indicators

If the health systems perform well in detecting and responding to cholera, the main predisposing factors then fall under other sectors. Actions on main drivers of cholera are not under health systems and require a global development multisectoral approach mainly on access to WASH. Our analysis confirms correlations and relationships between WASH indicators and the occurrence of cholera outbreaks. Risks factors include drinking directly from unprotected water sources, limited hygiene facilities, lack of soap and/or water, and open defaecation. This finding confirms the explanation of Gavi on why cholera continues to threaten many African countries.^19^ Gavi opined that cholera outbreaks have persisted in Africa because of worsening sanitation, poor and unreliable water supplies and worsening socioeconomic conditions. In other efforts, UNICEF^20^ in 2024 has reported that cholera epidemics that affected multiple countries in eastern and southern Africa in 2023 have persisted because of poor sanitation and hygiene infrastructures in the countries. A recent review has also attributed the deteriorating cholera situation in the African region to pervasive poor socioeconomic development which also has an impact on the provision of essential infrastructure for controlling and interrupting the outbreaks.^21^

The analysis also revealed substantial challenges in access to basic WASH facilities in the African region, with the population of 1.43 billion in 2022. This is like outcomes in other studies, which have also enumerated key drivers as inequitable access to such essentials as water, sanitation, education, good housing, and healthcare on the continent.^22^,^26^ Based on the current trend, the SDG targets for 2030 for water and sanitation, as many other SDGs, will not be reached, putting the region at risk for the cholera elimination objective by 2030.^27^ The rate of change in improving access to basic potable water and improved sanitation services is too slow to enable universal access to safe water, hygiene services and improved sanitation. Over the access to sanitation, cultural beliefs remain strong to enable open defaecation elimination.^27^

### About new commitment to cholera elimination

The findings of this study call for more commitment to cholera in the region. Access to WASH remains the main driver for cholera outbreaks in the region, calling on political leaders and governments to invest more in WASH structures and services. This investment will be a cost-effective investment that will have an impact not only on cholera but other diarrhoeal and water-borne diseases. The political commitment of some leaders to cholera elimination should be followed by adequate funding support for the implementation of the regional framework for cholera elimination by 2030. A recent assessment of the implementation of this framework reveals that only three countries are on track for disease elimination by 2030; this study also reveals the need to improve the availability of needed tools for cholera elimination such as vaccines and innovative laboratory confirmation devices.^18^

The implementation of the regional framework for cholera elimination by 2030 needs to be urgently improved and key milestones implemented. To this end, cholera technical teams should understand that strengthening cholera preparedness in hotspots and in known cholera prone countries will not be sufficient. All countries should prioritise cholera as a major threat, ensure its inclusion in surveillance guidelines, and more importantly strengthen preparedness and readiness in 100% of health districts. Cholera response structures should further include a strong preparedness component that will prevent the spread of the outbreaks to new areas.

## Conclusion

Insufficient access to safe WASH remains the main predisposing factor for cholera in the WHO African region. The region is not on track to have sustainably managed water resources, provide basic sanitation, and eliminate open defaecation by 2030. Alarming trends are evident when considering the access to WASH conditions as well as other operational and political issues. Urgent and targeted interventions are imperative to address these critical disparities and guide the region toward meeting its sanitation and water goals. Political leaders should take adequate actions to improve access to WASH and to strengthen multisectoral collaboration. More roles should be given to cities’ mayors in coordinating prevention and responses to cholera in their areas. Ministries of health should improve availability of adequate infectious disease management structures to be able to ensure adequate fulfilment of infection prevention and control principles; and strengthen health services for quick control of cholera outbreaks. Communities should commit and engage for cholera elimination and take the lead for adequate action on drivers in their areas. Although progress is being reported on some milestones for countries’ implementation of a framework for control,^26^ we argue that more needs to be done. Availability of vaccines and other tools, and sovereignty of them in the region, should be strengthened under the lead of WHO and the Africa Centres for Disease Control and Prevention, to enable availability of needed tools for adequate prevention and control of cholera outbreaks.

## supplementary material

10.1136/bmjgh-2024-016491 online supplemental file 1

## Data Availability

Data are available in a public, open access repository.
