# Supplementary material for: Exploring the burden of cholera in the WHO African region: patterns and trends from 2000 to 2023 cholera outbreak data
Source: BMJ Glob Health. 2025 Jan 22;10(1):e016491. doi: 10.1136/bmjgh-2024-016491 (PMC11891530; doi:10.1136/bmjgh-2024-016491)
Supplement: online supplemental file 1 [file bmjgh-10-1-s001.pdf]

SUPPLEMENTARY MATERIALS

Table 1. Statistics of cholera outbreaks from 2000 to 2023

Year	# Outbreaks	# Countries	Total cases	Total deaths	CFR
2000	25	25	115,160	4,365	3.8
2001	31	26	171,524	2,466	1.4
2002	34	26	135,091	4,392	3.3
2003	34	28	97,047	1,828	1.9
2004	41	30	91,070	2,305	2.5
2005	44	31	125,082	2,230	1.8
2006	39	31	203,564	5,281	2.6
2007	33	31	110,837	2,287	2.1
2008	43	34	160,735	4,940	3.1
2009	31	28	203,444	4,828	2.4
2010	26	21	109,549	3,275	3.0
2011	28	25	110,915	3,052	2.8
2012	33	26	95,534	1,842	1.9
2013	22	21	49,465	1,226	2.5
2014	19	18	102,467	1,882	1.8
2015	20	15	63,640	853	1.3
2016	20	16	55,439	1,214	2.2
2017	22	13	104,421	2,213	2.1
2018	36	16	113,889	2,391	2.1
2019	23	14	51,641	857	1.7
2020	13	12	40,835	697	1.7
2021	22	20	137,125	4,057	3.0
2022	26	17	84,831	1,867	2.2
2023	19	17	193,867	2,834	1.5
**2000-2023**	**684**	**44**	**2,727,172**	**63,182**	**2.3**

Table 2. Most affected countries based on cumulative cases and deaths 2000 -2023

	# outbreaks	Total cases	Total case rate per 100,000	Total deaths	Total death rate per 100,000	CFR
Dem Rep Congo	24	588,996	576.0	13,877	13.6	2.36
Nigeria	29	358,774	160.3	10,817	4.8	3.01
Mozambique	22	212,385	626.6	1,658	4.9	0.78
Zimbabwe	25	163,694	982.2	6,612	39.7	4.04
South Africa	13	161,744	267.7	566	0.9	0.35
Ethiopia	5	161,490	127.6	1,997	1.6	1.24
Angola	11	112,038	305.4	3,964	10.8	3.54
Malawi	18	104,712	500.3	2,724	13.0	2.60
Tanzania	19	98,034	145.4	2,036	3.0	2.08
Cameroon	11	76,724	267.8	2,822	9.9	3.68
Ghana	14	72,740	213.2	978	2.9	1.34
Kenya	24	71,121	129.1	1,373	2.5	1.93
Liberia	7	57,772	1,066.2	99	1.8	0.17
Zambia	18	55,030	267.5	1,559	7.6	2.83
Uganda	30	46,826	96.4	1,295	2.7	2.77
Guinea-Bissau	5	44,964	2,090.5	695	32.3	1.55
Senegal	7	38,591	217.3	522	2.9	1.35
Chad	8	37,676	206.1	1,319	7.2	3.50
Madagascar	2	36,556	120.5	2,085	6.9	5.70
South Sudan	5	30,526	275.3	652	5.9	2.14
Sierra Leone	3	28,861	328.3	557	6.3	1.93
Niger	18	27,479	101.0	925	3.4	3.37
Guinea	11	26,331	185.6	1,017	7.2	3.86
Burundi	23	14,928	112.8	165	1.2	1.11
Benin	14	14,164	103.3	210	1.5	1.48
Cote d'Ivoire	7	13,931	48.2	595	2.1	4.27
Congo	9	11,923	195.2	447	7.3	3.75
Mali	6	10,076	43.3	587	2.5	5.83
Togo	10	8,622	95.2	297	3.3	3.44
Eswatini	4	7,098	586.2	154	12.7	2.17
Comoros	3	6,706	787.0	170	20.0	2.54
Equatorial Guinea	1	6,450	376.2	34	2.0	0.53
Rwanda	4	4,489	31.8	52	0.4	1.16
Namibia	5	4,342	166.7	59	2.3	1.36
Mauritania	1	4,320	88.8	81	1.7	1.88
Sao Tome & Principe	2	3,101	1,337.5	46	19.8	1.48
Burkina Faso	6	2,315	10.0	46	0.2	1.99
Gabon	3	637	26.1	-	-	0.00
Central African Rep	2	458	8.0	63	1.1	13.76
Gambia	2	227	8.2	14	0.5	6.17
Algeria	1	217	0.5	2	0.0	0.92
Seychelles	1	178	165.3	1	0.9	0.56
Eritrea	2	120	3.2	9	0.2	7.50
Botswana	2	23	0.9	3	0.1	13.04

Table 3. Water, Sanitation and Hygiene indicators

**Water**	***Safely managed***	***Basic***	***Limited***	***Unimproved***	***Surface water***
	Drinking water from an improved water source that is accessible on premises, available when needed and free from faecal and priority chemical contamination	Drinking water from an improved source, provided collection time is not more than 30 minutes for a roundtrip including queuing	Drinking water from an improved source for which collection time exceeds 30 minutes for a roundtrip including queuing	Drinking water from an unprotected dug well or unprotected spring	Drinking water directly from a river, dam, lake, pond, stream, canal or irrigation canal
**Sanitation**	***Safely managed***	***Basic***	***Limited***	***Unimproved***	***Open defecation***
	Use of improved facilities that are not shared with other households and where excreta are safely disposed of in situ or removed and treated offsite	Use of improved facilities which are not shared with other households	Use of improved facilities shared between two or more households	Use of pit latrines without a slab or platform, hanging latrines or bucket latrines	Disposal of human faeces in fields, forests, bushes, open bodies of water, beaches and other open spaces or with solid waste
**Hygiene**	***Basic***	***Limited***	***No facility***		
	Availability of a handwashing facility with soap and water at home	Availability of a handwashing facility lacking soap and/or water at home	No handwashing facility on premises		

Table 4: Correlation between water, sanitation and hygiene coverage and cholera disease burden, in WHO African region, 2023.

WaSH factor	Correlation	P-value
Sanitation-basic	-0.27	>0.05
Sanitation-limited	0.17	>0.05
Hygiene-no-facility	0.04	>0.05
Surface-water	0.32	<0.05
Water-unimproved	0.27	>0.05
Sanitation-open-defecation	0.1	>0.05
Water-limited	0.31	<0.05
Sanitation-unimproved	0.26	>0.05
Hygiene-basic	0.31	<0.05
Hygiene-limited	0.37	<0.05
Water-basic	-0.39	<0.05

Table 5. IDSR reporting on cholera by country since the last revision of IDSR

Country	2019	2020	2021	2022	2023
Benin	*	*	*	*	*
Botswana	*	*	*	*	*
Burkina Faso	*	*	*	*	*
Burundi	*	*	*	*	*
Cameroon	*		*	*	*
Cabo Verde	*	*	*	*	*
CAR		*			
Chad	*	*	*	*	*
Congo	*				*
Cote d'Ivoire	*		*	*	*
DRC	*	*	*	*	*
Eritrea					*
Gabon	*	*	*	*	*
Gambia		*	*	*	*
Ghana	*	*	*	*	*
Guinea	*	*	*	*	*
Guinea-Bissau				*	*
Kenya				*	
Lesotho				*	
Liberia	*	*	*	*	
Madagascar	*	*	*	*	*
Malawi	*	*		*	
Mali	*	*	*	*	*
Mauritania					*
Mozambique	*	*	*	*	*
Namiba	*	*	*	*	*
Niger	*	*		*	*
Nigeria	*	*	*	*	*
Rwanda				*	
Sao Tome & Principe	*	*	*	*	*
Senegal	*	*	*	*	*
Seychelles	*	*			*
Sierra Leone	*	*	*	*	*
South Sudan	*	*	*	*	*
Togo	*	*	*	*	*
Uganda				*	*
United Rep. of Tanzania	*	*	*	*	*
Zambia	*	*	*	*	*
Zimbabwe	*	*	*	*	*

Table 6. Measuring time to detection and time to contain a cholera outbreak (2015-2023 data)

		Time to detection		Time to control		
**Year**	**# outbreaks**	**Included**	**median (IQR)**	**Closed**	**included**	**median (IQR)**
2015	2	0		2	1	1,054 (1,054 - 1,054)
2016	3	3	68 (49 - 227)	3	2	418 (327 - 508)
2017	13	9	3 (0 - 5)	13	10	134 (120 - 205)
2018	22	16	1 (0 - 8)	22	17	49 (25 - 86)
2019	17	13	0 (0 - 3)	17	17	89 (26 - 203)
2020	7	5	4 (0 - 5)	7	5	131 (61 - 233)
2021	13	12	2 (0 - 6)	12	10	84 (50 - 108)
2022	17	16	1 (0 - 6)	12	10	88 (46 - 161)
2023	13	10	4 (2 - 7)	6	6	163 (113 - 181)
2024	1	1	4 (2 - 7)	0	0	
Missing	2	0		2	0	
Overall	110	85	2 (0 - 7)	96	78	92 (46 - 181)

Table 7. Measuring time to detection and time to contain a cholera outbreak by country (2015-2023 data)

		Time to detection		Time to control		
**Country**	**# outbreaks**	**Included**	**median (IQR)**	**Closed**	**included**	**median (IQR)**
Algeria	1	1	17 (17 - 17)	1	0	
Angola	6	2	29 (28 - 29)	6	5	115 (41 - 220)
Benin	3	2	2 (1 - 2)	3	3	233 (161 - 248)
Burkina Faso	2	2	1 (0 - 1)	2	2	48 (26 - 69)
Burundi	7	7	0 (0 - 3)	6	4	83 (22 - 142)
Cameroon	3	3	0 (0 - 2)	2	2	534 (471 - 594)
Cape Verde	1	1	5 (5 - 5)	1	1	8 (8 - 8)
Chad	2	2	2 (1 - 2)	2	2	101 (93 - 110)
Congo	2	1	0 (0 - 0)	2	1	100 (100 - 100)
DRC	2	1	6 (6 - 6)	1	1	174 (174 - 174)
Eswatini	1	1	2 (2 - 2)	1	1	65 (65 - 65)
Ethiopia	3	3	0 (0 - 8)	2	1	753 (753 - 753)
Ghana	1	1	12 (12 - 12)	1	1	20 (20 - 20)
Kenya	6	4	0 (0 - 0)	5	5	77 (73 - 604)
Malawi	7	5	4 (0 - 4)	6	4	74 (47 - 114)
Mali	1	1	3 (3 - 3)	1	1	60 (60 - 60)
Mozambique	7	6	2 (0 - 32)	6	5	260 (90 - 338)
Namibia	1	0		1	1	6 (6 - 6)
Niger	3	3	7 (6 - 9)	3	2	198 (181 - 214)
Nigeria	6	2	19 (9 - 28)	5	4	178 (139 - 313)
South Africa	4	3	1 (1 - 2)	3	3	48 (37 - 116)
South Sudan	4	4	7 (5 - 22)	3	3	244 (210 - 422)
United Rep. Tanzania	8	4	7 (5 - 8)	7	5	118 (114 - 191)
Togo	3	3	2 (1 - 4)	2	2	54 (51 - 58)
Uganda	11	10	3 (0 - 5)	11	10	82 (38 - 119)
Zambia	9	7	4 (1 - 23)	8	6	21 (14 - 66)
Zimbabwe	6	6	0 (0 - 2)	5	3	54 (31 - 56)
Overall	110	85	2 (0 - 7)	96	78	92 (46 - 181)
